# Impact of Respiratory Support During Hospitalization on Functional Outcomes in Long COVID: A Post-Hoc Analysis of a Prospective Cohort Study

**DOI:** 10.3390/ijerph22010049

**Published:** 2024-12-31

**Authors:** Camila Miriam Suemi Sato Barros do Amaral, Jefferson Valente, Cássia da Luz Goulart, Bernardo Maia da Silva, Alexandre Silva Neto, Nadia Cubas-Vega, Anna Gabriela Rezende, Eduardo Fernandes, Mayla Gabriela Silva Borba, Vanderson Sampaio, Wuelton Monteiro, Gisely Cardoso de Melo, Marcus Lacerda, Guilherme Peixoto Tinoco Arêas, Fernando Almeida-Val

**Affiliations:** 1 Fundação de Medicina Tropical Dr Heitor Vieira Dourado, Manaus 69040-000, Brazil; milasuemi@yahoo.com.br (C.M.S.S.B.d.A.); cassiadaluzgoulart@gmail.com (C.d.L.G.); bernardo.mpesq88@gmail.com (B.M.d.S.); alexandre.neto94@gmail.com (A.S.N.); wueltonmm@gmail.com (W.M.); cardosogisely@gmail.com (G.C.d.M.); marcuslacerda.br@gmail.com (M.L.); 2 Universidade do Estado do Amazonas, Manaus 69065-001, Brazil; jefferson.valente@yahoo.com.br (J.V.); eduardo.fernandes@me.com (E.F.); maylagsborba@gmail.com (M.G.S.B.); 3 Universidad Nacional Autónoma de Honduras, Tegucigalpa 11101, Honduras; nadiadr13@gmail.com; 4 Universidade Federal do Amazonas, Manaus 69067-005, Brazil; annagabrielarezende@gmail.com (A.G.R.); guilhermepta@ufam.edu.br (G.P.T.A.); 5 Hospital e Pronto-Socorro Delphina Rinaldi Abdel Aziz, Manaus 69093-415, Brazil; 6 Instituto Todos pela Saúde, São Paulo 01310-942, Brazil; vandersons@gmail.com; 7 Instituto Leônidas & Maria Deane, Fundação Oswaldo Cruz (ILMD/Fiocruz Amazônia), Manaus 69057-070, Brazil; 8 Fernando Fonseca de Almeida e Val, Fundação de Medicina Tropical Doutor Heitor Vieira Dourado, Av. Pedro Teixeira, 25 Bairro Dom Pedro, Manaus 69040-000, Brazil

**Keywords:** COVID-19, post-acute COVID syndrome, functionality, respiratory support, oxygen supplementation

## Abstract

Post-acute COVID-19 syndrome (PACS) is characterized by the persistence of one or more symptoms after the acute phase, leading to physical disabilities. This study aims to investigate whether the functional capacity and respiratory function 120 days post-COVID-19 differed according to the level of respiratory support needed during hospitalization in acute COVID-19 in the pre-vaccine rollout period. We followed up with 118 COVID-19 hospitalized patients in the acute phase until 120 days post-acute disease, with patients split into a Non-Invasive Oxygen Therapy Group (OTG, n = 72), Invasive Mechanical Ventilation Group (IMV, n = 12), and Room Air Group (RAG, n = 34), assessing the body composition, respiratory muscle strength, pulmonary function, functional capacity, and muscle strength at the follow-up visit. In total, 54 individuals (45.8%) were female, with a median age of 48 years old (IQR: 41–58). We found that the group with IMV was older (*p* < 0.001), had more admissions to the ICU (*p* < 0.001), and had longer hospital stays (*p* < 0.001). There were no statistically significant differences between groups (OTG, IMV, and RAG) for the spirometry function (*p* = 0.31), DASI score (*p* = 0.77), manovacuometry (MIP *p* = 0.74; MEP *p* = 0.23), 6MWT (*p* = 0.43), and handgrip (*p* = 0.19) outcomes. At D120, the IMV group had an important loss of body muscle mass (BMM) and a higher BMM than RAG (*p* = 0.02). Reduction in MIP (*p* = 0.01) and MEP (*p* = 0.02) in the IMV group and OTG group when compared to the RAG was also observed. Functional outcomes at 120 days from COVID-19 hospitalization were not found to be associated with the levels of oxygen supplementation during acute disease in this group of participants.

## 1. Introduction

The World Health Organization has defined Long COVID or Post-acute COVID-19 syndrome (PACS) as the presence, emergence, or worsening of symptoms, usually for at least 3 months from the onset of COVID-19, and lasting at least 2 months, without explanation by alternative diagnoses. PACS has a growing spectrum of long-term health consequences, following SARS-CoV-2 virus infection that is not yet fully understood [1], with a prevalence of 54% in hospitalized individuals and 34% in non-hospitalized individuals, an estimated global prevalence of 43% [2]. Female gender, pre-existing hypertension, and length of hospital stay were associated with an increased risk of new or persistent symptoms [1]. Some findings indicate prolonged illness even among persons with milder outpatient illness [3]. The management of PACS has been focused on treating symptoms without a unanimous approach, and, even if treating symptoms benefits patients in the short term, there is a need to identify factors involved in the pathological mechanisms of the disease [4].

The COVID-19 pandemic, caused by the SARS-CoV-2 virus, has led to widespread respiratory complications, requiring various forms of respiratory support ranging from Invasive mechanical ventilation (IMV) to non-invasive oxygen therapy (OT), and, in milder cases, room air (RA) management. The prognosis of post-COVID-19 patients is thought to be significantly influenced by the type and intensity of respiratory support received during the acute phase of the illness [5]. However, there remains a gap in our understanding of how these different interventions affect long-term respiratory function and functional capacity.

In a 6 month follow-up after acute infection, COVID-19 survivors who were more severely ill during their hospital stay had poorer pulmonary diffusion capacities and abnormal chest imaging manifestations [6]. Another study provided evidence of substantially greater neurological and psychiatric morbidity in patients who had severe COVID-19, but was not limited to them [7]. The persistence of atypical chronic symptoms after the acute phase of the disease includes extreme fatigue, post-exercise malaise, and shortness of breath independently of disease severity [8,9,10]. In a multicenter study, it was observed that individuals undergoing different types of oxygen therapy presented different levels of quality of life in the long term after COVID-19 [11].

The impact of different methods for respiratory support during severe COVID-19 on long-term functional outcomes is still a matter of debate. The guiding hypothesis of the study is “Are there expected differences in long-term functional capacity among patients receiving different levels of respiratory support?”. Therefore, this study aimed to investigate whether respiratory and functional capacity 120 days post-acute COVID-19 were different between those who received non-invasive oxygen therapy (OTG), invasive mechanical ventilation (IMV), and room air (RAG) during hospitalization, in Manaus, Western Brazilian Amazon.

## 2. Methods

### 2.1. Study Design and Population

**Figure 1:**
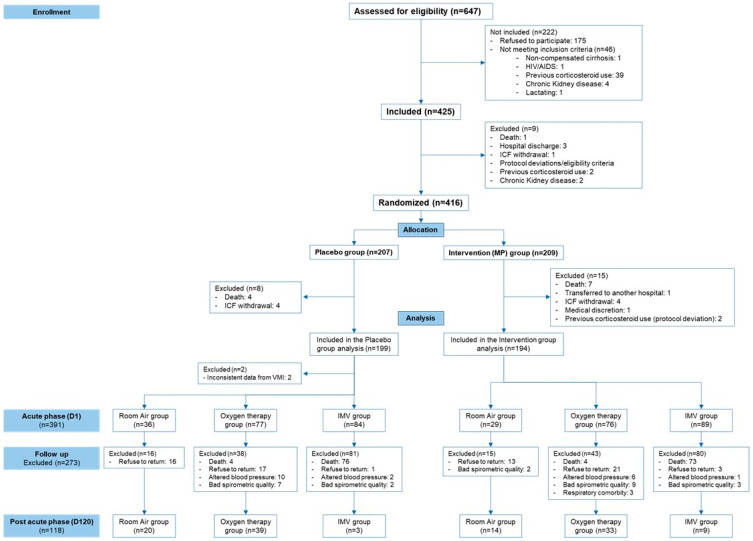
Study flowchart.

A post hoc analysis of prospectively collected data was conducted at the Hospital e Pronto-Socorro Delphina Rinaldi Abdel Aziz in Manaus, western Brazilian Amazon, from April to October 2020. COVID-19 patients were followed up until 120 days after hospital discharge. The 120-day period used here falls exactly within the time interval defined by WHO for PACS. The study flowchart is shown in Figure 1. Methods, eligibility criteria, and results of the clinical trial have already been published elsewhere [12,13]. Briefly, individuals aged 18 years or older, of both sexes, with SpO_2_ ≤ 94% on room air, requiring supplementary oxygen, or under IMV at the hospital were included in the clinical trial. For this study, participants were stratified into three groups according to the need for respiratory support during the hospitalization period (OTG, IMV, and RAG).

### 2.2. Study Assessments

Clinical and laboratory data from hospitalization were collected in an electronic medical recording system (Medview version 710801 and Esthor) and then registered in an electronic database (REDCap). The functional assessments were performed on D120. All procedures were performed according to test guidelines by trained and harmonized study staff.

### 2.3. Body Composition

Weight, body mass index (BMI), muscle mass (Kg), and fat mass (Kg) variables were extracted in the assessment of body composition. Body composition was measured with participants standing barefoot and upright on a calibrated scale (OMRON^®^, Kusatsu, Japan). Individuals were on a fasting regime at the time of assessment. A standard measuring tape divided into centimeters was used to collect the height of the participants, keeping them barefoot and with their bodies upright.

### 2.4. Pulmonary Function and Respiratory Muscle Strength (RMS)

A forced spirometry maneuver was performed using a spirometer (Cosmed^®^, Rome, Italy) to determine pulmonary function. Spirometry results (obstructive, restrictive, and normal) and the predicted values were defined by the ERS/American Thoracic Society (ATS) [14,15]. Maximum inspiratory pressure (MIP) and maximum expiratory pressure (MEP) assessments were performed to evaluate RMS using a digital manometer (MDI, MVD300, Bahia, Brazil), and the Neder equation was used to define the reference value [16]. Both tests were performed according to the American Thoracic Society and European Respiratory Society guidelines [17]. Those who presented altered blood pressure levels and those with poor-quality spirometry were not included in the analysis.

### 2.5. Functional Capacity

The six-minute walk test (6MWT) was performed to evaluate the participant’s functional capacity. The subjects were instructed with standardized verbal encouragement to walk the longest possible distance in six minutes. The test was performed on a 30 m long flat corridor according to the American Thoracic Society’s recommendations [18]. The predicted value for the Brazilian population was used to define the estimated distance for sex and age [19]. The Duke activity status index (DASI) was also performed. It is a standardized questionnaire consisting of a 12-item self-reported about usual physical activities correlated with gold-standard measures of functional capacity; the maximum best score is 58.2 [20,21].

### 2.6. Handgrip Strength (HGS)

Three measurements were performed in a standing position for each hand, with the elbow straight and fully extended, a 1 min interval, alternating between dominant and non-dominant sides. The maximum value was derived for each arm [22]. The HGS was performed using a digital hand-held dynamometer (Instrutherm^®^, São Paulo, Brazil).

### 2.7. Statistical Analysis

Mean and standard deviation (SD) were calculated when data distribution was normal. The Shapiro–Wilk test was used to verify data distribution between groups; for variables that were not included in the normal distribution, nonparametric tests were used (Mann–Whitney test for comparisons between two independent groups or Kruskal–Wallis test followed by comparisons for more than two groups). Categorical variables were analyzed using Pearson’s chi-square test to assess associations between two categorical variables when parametric, and Spearman’s test to assess associations between two categorical variables when non-parametric. Descriptive data are shown as a mean and standard deviation or median and interquartile range (IQR) when appropriate. The ANOVA two-away test was used to compare the OTG, IMV, and RAG groups with the post hoc of Tukey to perform multiple pairwise comparisons after ANOVA. Missing data were removed from the analysis, as the proportion of missing values was low and would not compromise the representativeness of the sample. All tests were performed using R software version 4.3.0 and *p*-values ≤ 0.05 were considered statistically significant.

### 2.8. Ethical Aspects

All hospitalized patients were informed about the study objectives and risks, and they were invited to participate. All individuals were given time to read and sign an informed consent form carefully. This study was conducted following the Declaration of Helsinki principles and the Good Clinical Practice guidelines of the International Conference on Harmonization. The Brazilian Committee on Ethics in Human Research approved the study (CAAE 30615920.2.0000.0005).

## 3. Results

A total of 118 participants with PACS were included in this analysis, of which 54 (45.8%) were female, with a median age of 48 (IQR: 41–58). We stratified patients using RAG (n = 34), OTG (n = 72), and IMV (n = 12) during acute COVID-19 hospitalization. The IMV group was found to be older (*p* < 0.001) with a predominance of males (*p* < 0.01), more ICU admissions (*p* < 0.01), longer hospital stay (*p* < 0.01), as well as an increase in leukocytes (*p* = 0.02), lower lymphocytes (*p* < 0.01), increased alanine aminotransferase (*p* = 0.001), CPK (*p* = 0.04), C-reactive protein (*p* < 0.001), and IL-6 (*p* < 0.001) when compared to the other groups.

There was no difference between the groups regarding using corticosteroid therapy during the acute phase of the disease (*p* = 0.12). The IMV group used more oseltamivir (Tamiflu^®^) (*p* < 0.001), insulin (*p* < 0.001), and more anticoagulants when compared to the other groups (*p* < 0.001). Clinical and laboratory data at the hospital presentation are presented in Table 1.

The IMV group had an average of 10 days (IQR: 5–14) under protective IMV (lower tidal volume, driving pressure ≤ 15 cm H_2_O, plateau pressure ≤ 30 cm H_2_O), with an average static lung compliance of 35.3 (IQR: 34.1–44.4) cm H_2_O. In the OTG group, 63 were on nasal catheters and 9 were on non-rebreather masks.

Even though the IMV group had a more severe clinical profile, including more elevated inflammatory markers, we did not observe any difference in respiratory function and functional capacity when compared to the oxygen therapy and air ambient groups at the 120 day follow-up visit (Table 2). The 6MWD in meters among all groups was similar (*p* = 0.31), corresponding to 71.7% of the predicted distance for the Brazilian population [23], but without difference between groups (*p* = 0.43). Similarly, the DASI score did not demonstrate any statistical significance (*p* = 0.77).

At D120, the IMV group had an important loss of body muscle mass (BMM), although it remained higher when compared with room air (*p* = 0.02). Reduction in MIP (*p* = 0.01) and MEP (*p* = 0.02) in the IMV group and OTG group when compared to the RAG was also observed. On the other hand, handgrip strength (*p* = 0.19) did not present a statistical difference between groups.

## 4. Discussion

This study demonstrated that despite being less severe in the acute phase, patients with COVID-19 who did not require IMV and remained on room air or only under oxygen therapy support had the same respiratory and functional capacity as critically ill patients who required IMV four months after hospitalization.

The association between the use of IMV and worse clinical outcomes is somewhat expected, which may include the use of anticoagulants and insulin and longer hospital stays. The need for IMV generally indicates more organ damage, with greater pulmonary and systemic tissue damage [24]. Furthermore, mechanical ventilation may contribute to ventilator-induced lung injury (VILI), exacerbating the inflammatory process and dysfunction of other organs [24,25]. It is worth noting that participants in the IMV group were mostly under protective ventilation, which may be associated with similar performance on functional capacity of this group, in the post-acute phase of COVID-19, of less severe patients.

The decrease in respiratory muscle strength is one of the complications observed in cases of COVID-19 [26,27,28,29]. Here, we found that the IMV group presented a significant decrease in these parameters (MIP and MEP), which can occur for some reasons, from systemic inflammation caused by SARS-CoV-2 infection, which can affect the respiratory muscles, to prolonged immobilization, which can lead to muscle atrophy.

Individuals in the IMV group had higher body muscle mass compared to the other groups in the admission, which may be a characteristic restricted to the study population, since the literature shows that most critically ill patients lose muscle because of an inability to maintain protein synthesis rates and show a decline in muscle health [30,31,32]. When evaluated on D120, even though they continued to have higher body muscle mass values, the IMV group showed the greatest drop in these values (Δ21.5 kg), showing that individuals lost a lot of mass during hospitalization, which is in line with the literature [30]. The decrease in lean muscle mass observed in the IMV group may be a consequence of prolonged immobilization, stress-induced catabolism, and systemic inflammation [33]; loss of muscle mass is associated with increased morbidity and mortality in critically ill patients [34]. We believe that because they had greater lean mass upon admission, they were not as impacted as the other groups and were able to perform the functional assessments similarly to the less severe patients.

The loss of muscle mass in COVID-19 patients admitted to ICU during acute disease has already been observed before [35], and its persistence has been reported 15 months after hospitalization [36]. Histopathological changes with atrophy and degeneration in addition to mitochondrial changes may be present and justify the fatigue symptoms of PACS [37]. Reduced spirometry and functional parameters are associated with post-COVID patients with reduced handgrip strength [38]. In this study, handgrip strength was equal between groups, but respiratory muscle strength was worse in the IMV group, which had greater loss of muscle mass and longer hospital stays.

Huang et al. (2020) [5] observed that severe COVID-19 patients had a 6MWD decline when compared with non-severe cases. In Eksombatchai et al. (2021) [39] the mean 6MWD compared in mild symptom patients vs. non-severe pneumonia COVID-19 patients and evidenced a not significant difference (*p* = 0.11), but most abnormal spirometry cases were in the severe pneumonia group (*p* = 0.001). In our study, it was not possible to identify any statistical difference between groups at the 4-month follow-up visit, and all spirometry values were within the normal range.

During the chaotic first pandemic peak of COVID-19, some therapies, such as non-invasive ventilation, were not recommended due to the difficulty in accessing resources. Many patients received IMV or delayed receiving other advanced life support due to the lack of resources secondary to the collapse of health services at the time, which resulted in a large sample loss and may have influenced the findings in survivors on D120. Furthermore, no data were collected on the daily habits and lifestyle of the participants between D28 and D120.

Some limitations include the fact that there was no assessment of functional outcomes at hospitalization discharge, which impairs longitudinal comparisons. Also, the slow vital capacity of the participants was not performed. This was a single-center study and may present results that cannot be generalized to other populations or contexts. The study’s follow-up period was relatively short, which makes it difficult to assess the durability of the effects and monitor chronic conditions or the emergence of new symptoms. Another limitation of the study is that the groups are unbalanced in size, gender, and BMM. Furthermore, all assessments were performed in individuals infected by SARS-CoV-2 in the early- and mid-2020s; therefore, extrapolation of outcomes to individuals infected with more recent variants, or reinfections, may not be performed. Nonetheless, a great number of those infected before the vaccine rollout have debilitating PACS currently, and, therefore, the results presented here may help to manage this population.

## 5. Conclusions

In conclusion, the results presented here may help to evidence that individuals who presented severe COVID-19 in early- and mid-2020, with different needs for respiratory support during hospitalization, presented some similar functional outcomes after discharge. Individuals undergoing IMV had an important loss of body muscle mass and reduction in MIP and MEP, which can also be attributed to hospitalization. This reinforces the finding that the use of respiratory support during hospitalization for severe COVID-19 may not be a detrimental factor for the development of functional abnormalities months after hospital discharge. By understanding the factors that influence long-term outcomes in COVID-19 survivors, healthcare providers and policymakers can work together to provide the necessary support and resources to help individuals recover and regain their quality of life. Given the potential for delayed or persistent symptoms, healthcare systems should establish long-term monitoring programs to identify and address ongoing health issues in COVID-19 survivors. This may involve regular check-ups with healthcare providers, including primary care physicians and specialists, as well as access to rehabilitation services as needed.

## Figures and Tables

**Table 1:** Clinical characteristics of patients at hospital presentation.

Variable	Total	Room Air	Oxygen Therapy	IMV	*p*-Value
	N = 118	N = 34	N = 72	N = 12	
Baseline characteristics					
Age, Years, mean (SD)	49.3 (13.2)	42.1 (9.3) *	51.9 (14.0)	54.2 (10.3)	**<0.001**
Gender (female), n/N (%)	54/118 (45.8%)	24/34 (70.6%) *	28/72 (38.9%)	2/12 (16.7%) #	**<0.001**
BMI, kg/m^2^, mean (SD)	31.5 (6.7)	31.1 (7.5)	31.9 (6.6)	30.9 (5.3)	0.79
BMM, kg, median (IQR)	26.2 (18.8–34.6)	20.9 (16.5–24.8)	28.8 (21.0–42.8)	49.8 (32.4–54.2) #	**<0.001**
BFM, kg, median (IQR)	28.3 (22.1–42.1)	33.8 (22.5–44.5)	28.0 (22.1–41.6)	27.0 (17.5–34.6)	0.57
Previous coexisting disease					
Comorbidities, n/N (%)	112/118 (94.9%)	31/34 (91.2%)	69/72 (95.8%)	12/12 (100.0%)	0.42
Smoking history, n/N (%)	2/112 (1.8%)	1/31 (3.2%)	0/69 (0.0%)	1/12 (8.3%)	0.10
Diabetes, n/N (%)	30/112 (26.8%)	4/31 (12.9%)	22/69 (31.9%)	4/12 (33.3%)	0.12
Hypertension, n/N (%)	43/112 (38.4%)	10/31 (32.3%)	29/69 (42.0%)	4/12 (33.3%)	0.60
Alcohol user, n/N (%)	33/112 (29.5%)	8/31 (25.8%)	20/69 (29.0%)	5/12 (41.7%)	0.59
Chronic heart disease, n/N (%)	7/112 (6.3%)	1/31 (3.2%)	3/69 (4.3%)	3/12 (25.0%) #†	**0.01**
Chronic lung disease, n/N (%)	5/112 (4.5%)	3/31 (9.7%)	1/69 (1.4%)	1/12 (8.3%)	0.14
Chronic rheumatic disease, n/N (%)	10/112 (8.9%)	2/31 (6.5%)	7/69 (10.1%)	1/12 (8.3%)	0.83
Chronic liver disease, n/N (%)	13/112 (11.6%)	6/31 (19.4%)	7/69 (10.1%)	0/12 (0.0%)	0.17
Tuberculosis, n/N (%)	3/112 (2.7%)	2/31 (6.5%)	1/69 (1.4%)	0/12 (0.0%)	0.30
COPD, n/N (%)	1/112 (0.9%)	0/31 (0.0%)	1/69 (1.4%)	0/12 (0.0%)	0.73
Drugs used in the acute phase					
Methylprednisolone n/N (%)	56/118 (47.5%)	14/34 (41.2%)	33/72 (45.8%)	9/12 (75.0%)	0.12
Oseltamivir n/N (%)	66/114 (57.9%)	7/30 (23.3%)	50/72 (69.4%)	9/12 (75.0%) #†	**<0.001**
Insulin n/N (%)	41/108 (38.0%)	3/24 (12.5%)	31/72 (43.1%)	7/12 (58.3%) #†	**<0.001**
Anticoagulant n/N (%)	70/108 (64.8%)	7/24 (29.2%)	52/72 (72.2%)	11/12 (91.7%) #†	**<0.001**
Clinical and laboratory characteristics					
ICU on admission, n/N (%)	6/94 (6.4%)	0/11 (0.0%)	0/72 (0.0%)	6/11 (54.5%) #†	**<0.001**
IMV on admission, n/N (%)	6/118 (5.1%)	0/34 (0.0%)	0/72 (0.0%)	6/12 (50.0%) #†	**<0.001**
Oxygen saturation (SpO_2_), n/N (%)	97.0 (95.0–98.0)	98.0 (97.0–99.0)	96.0 (95.0–97.5)	95.0 (91.0–97.5)	**<0.001**
Blood culture, n/N (%)					**0.01**
Negative	72/89 (80.9%)	10/11 (90.9%)	55/70 (78.6%)	7/8 (87.5%)	
Positive	1/89 (1.1%)	0/11 (0.0%)	0/70 (0.0%)	1/8 (12.5%) #†	
Leukocytes, mm^3^, median (IQR)	9.5 (7.3–12.2)	9.5 (7.7–11.7)	9.2 (7.0–11.8)	13.2 (9.3–18.9) #†	**0.02**
Hemoglobin, g/dL, median (IQR)	12.6 (11.9–13.4)	12.6 (11.9–13.3)	12.5 (11.8–13.5)	13.4 (11.5–14.6)	0.34
Lymphocytes, mm^3^, mean (SD)	15.9 (8.8)	20.9 (9.2)	14.8 (7.6)	8.6 (7.2) #†	**<0.001**
Platelet count, 10 3/mm^3^, median (IQR)	308.5 (220.5–371.5)	297.0 (220.0–370.0)	313.0 (222.0–374.0)	315.0 (229.0–360.5)	0.96
Blood glucose, mg/dL, median (IQR)	163.0 (128.0–209.0)	158.0 (108.0–177.0)	169.5 (134.0–221.5)	164.0 (127.0–216.0)	0.35
Alanine aminotransferase, U/L, median (IQR)	56.1 (36.5–82.2)	35.8 (27.5–57.6) *	61.5 (42.5–93.0)	76.6 (55.9–80.0) #	**<0.001**
Creatinine, mg/dL, median (IQR)	0.9 (0.7–1.0)	0.9 (0.7–1.0)	0.8 (0.7–1.0)	1.1 (0.8–2.2)	0.15
CPK, U/L, median (IQR)	76.3 (39.9–144.1)	53.5 (30.2–128.6)	80.4 (42.2–134.6)	156.9 (73.4–306.0) #†	**0.04**
CK-MB, U/L, median (IQR)	18.2 (12.0–23.1)	14.7 (10.7–19.7)	18.7 (14.9–23.7)	19.4 (14.4–23.6)	0.08
Lactate dehydrogenase, U/L, mean (SD)	625.3 (352.4)	319.3 (113.1) *	639.3 (361.0)	861.2 (212.7) #	**0.01**
C-reactive protein, mg/L, median (IQR)	66.7 (29.1–87.3)	6.7 (6.3–53.6) *	67.7 (29.1–87.3)	77.6 (66.7–101.1) #	**<0.001**
IL-6, pg/mL, median (IQR)	31.8 (8.3–82.2)	5.8 (0.0–77.5) *	25.8 (9.0–68.9)	82.2 (36.4–232.4) #†	**<0.001**
qSOFA score ≥ 2, n/N (%)	16/118 (13.6%)	0/34 (0.0%)	12/72 (16.7%)	4/12 (33.3%) #†	**<0.001**
Computed Tomography					
Ground-glass opacity infiltration, n/N (%)	84/89 (94.4%)	11/11 (100.0%)	62/67 (92.5%)	11/11 (100.0%)	0.42
Consolidation, n/N (%)	70/89 (78.7%)	7/11 (63.6%)	53/67 (79.1%)	10/11 (90.9%)	0.29
Unilateral consolidation, n/N (%)	7/89 (7.9%)	2/11 (18.2%)	4/67 (6.0%)	1/11 (9.1%)	0.37
Bilateral consolidation, n/N (%)	72/89 (80.9%)	8/11 (72.7%)	54/67 (80.6%)	10/11 (90.9%)	0.55
Pleural effusion, n/N (%)	18/89 (20.2%)	2/11 (18.2%)	14/67 (20.9%)	2/11 (18.2%)	0.96
Hospitalization Data					
Length of hospital stay (Days), median (IQR)	9.0 (7.0–13.0)	7.0 (3.5–8.5)	9.0 (7.0–12.0)	20.5 (19.5–23.5) #†	**<0.001**
Time at VMI (Days), median (IQR)	10.0 (5.0–14.0)	NA	NA	10.0 (5.0–14.0)	
ICU length of stay (Days), median (IQR)	8.0 (5.5–15.0)	NA	1.0 (1.0–1.0)	9.0 (6.0–20.0)	0.13

* *p* < 0.05 Oxygen therapy versus ambient air; # *p* < 0.05 IMV versus ambient air; † *p* < 0.05 Oxygen therapy versus IMV; *p*-values with statistically significant are bolded. SD standard deviation, ICU intensive care unit, IMV invasive mechanical ventilation, BMI body mass index, BFM body fat mass, BMM body muscle mass, kg kilogram, IQR interquartile range, CPK creatine phosphokinase, CK-MB myoglobin creatine phosphokinase, IL Interleukin.

**Table 2:** Functional assessment in post-acute COVID-19 phase at 120 days follow up.

Variable	Total	RAG	OT	IMV	*p*-Value
	N = 118	N = 34	N = 72	N = 12	
Bioimpedance					
Weight (kg), mean (SD)	87.0 (19.9)	81.9 (20.5)	89.7 (20.5)	85.5 (11.1)	0.17
Muscle mass (kg), median (IQR)	23.9 (18.0–29.3)	21.2 (16.5–26.1) *	25.1 (18.4–30.1)	27.3 (22.1–29.5)	**0.02**
Fat mass (kg), median (IQR)	31.5 (25.0–41.2)	33.0 (24.9–43.6)	32.2 (26.7–41.2)	25.5 (22.2–33.8)	0.24
Spirometry					
FVC (L), mean (SD)	3.0 (0.8)	3.1 (0.9)	3.0 (0.8)	3.1 (0.7)	0.87
% FVC, mean (SD)	89.1 (20.3)	93.4 (18)	87.6 (22)	85.8 (13.8)	0.33
FEV1 (L), mean (SD)	2.4 (0.8)	2.5 (0.8)	2.4 (0.8)	2.5 (0.6)	0.59
%FEV1, mean (SD)	87 (21.5)	89.8 (22)	85.8 (22.5)	86.9 (12.6)	0.67
FEV1/FVC (%), median (IQR)	82.8 (77.5–85.4)	83.4 (80.5–85.5)	81.9 (75.6–85.3)	81.7 (78.5–85.4)	0.44
Spirometry interpretation (%)					0.81
Normal, n/N (%)	93/118 (78.8%)	27/34 (79.4%)	56/72 (77.8%)	10/12 (83.3%)	
Obstructive, n/N (%)	10/118 (8.5%)	3/34 (8.8%)	7/72 (9.7%)	0/12 (0.0%)	
Restrictive, n/N (%)	15/118 (12.7%)	4/34 (11.8%)	9/72 (12.5%)	2/12 (16.7%)	
Manovacuometry					
MIP (cmH_2_O), mean (SD)	93.9 (32.3)	90.4 (35.3)	95.2 (32.5)	96.8 (22.1)	0.74
% of predicted MIP (%), median (IQR)	79.9 (57.8–145.6)	100.8 (73.8–161.5)	76.3 (55.5–145.6) *	65.9 (58.8–72.7) #	**0.01**
MEP (cmH_2_O), mean (SD)	107.8 (35.5)	99.1 (38.6)	111.0 (35.4)	113.9 (23.6)	0.23
% of predicted MEP (%), median (IQR)	90.1 (72.3–171.5)	117.8 (88.6–175.9)	87.9 (69.6–171.5) *	74.7 (67.3–87.9) #	0.02
6MWT					
Walk distance (m), median (IQR)	390.0 (324.0–456.0)	411.0 (337.0–477.0)	380.0 (324.0–456.0)	353.0(283.0–431.0)	0.31
Percentage of predicted (%), mean (SD)	71.7 (15.4)	70.3 (16.9)	73.1 (14.6)	67.2 (15.6)	0.43
DASI Score	45.5 (30.2–50.7)	50.7 (28.7–53.0)	43.8 (31.5–50.7)	43.8 (29.1–58.2)	0.77
Handgrip					
Handgrip Test, Kgf, median (IQR)	30.2 (24.0–39.8)	26.8 (22.8–34.2)	31.7 (24.2–42.8)	31.3 (23.6–38.3)	0.19

* *p* < 0.05 Oxygen therapy versus ambient air; # *p* < 0.05 IMV versus ambient air; *p*-values with statistically significant are bolded. IMV: invasive mechanical ventilation; FVC: forced vital capacity; FEV: forced expiratory volume in the first second; MIP: maximal inspiratory pressure; MEP: maximal expiratory pressure; 6MWT: six-minute walk test; DASI: Duke activity status index.

## Data Availability

The original contributions presented in the study are included in the article. The raw data supporting the conclusions of this article shall be made available upon reasonable request to the corresponding author.
